# Patient-related risk factors of prosthetic joint infections following total hip and knee arthroplasty at King Abdulaziz Medical City, a 10-year retrospective study

**DOI:** 10.1186/s13018-023-04210-9

**Published:** 2023-09-22

**Authors:** Wazzan S. Aljuhani, Abdullah M. Alanazi, Abdullah I. Saeed, Khalid H. Alhadlaq, Yazeed S. Alhoshan, Ziad A. Aljaafri

**Affiliations:** 1 Department of Orthopedic Surgery, Ministry of the National Guard – Health Affairs, Riyadh, Saudi Arabia; 2 King Abdullah International Medical Research Center, Riyadh, Saudi Arabia; 3 King Saud Bin Abdulaziz University for Health Sciences, Riyadh, Saudi Arabia

**Keywords:** Total joint arthroplasty, PJI, Complications, Total knee arthroplasty, Total hip arthroplasty

## Abstract

**Background:**

Total joint arthroplasty (TJA) can be associated with the development of periprosthetic joint infection (PJI). It is necessary to determine the modifiable and non-modifiable risk factors of PJI to provide optimum healthcare to TJA candidates.

**Methods:**

This single-center retrospective review investigated 1198 patients who underwent TJA from 2012 to 2022. The data analysis comprised two stages. The first stage was a descriptive analysis, while the second stage was a bivariate analysis. The sociodemographic data, medical history, operative details, and presence of PJI postoperatively were evaluated.

**Results:**

The study sample consisted of 1198 patients who underwent TJA. The mean patient age was 63 years. Among the patients, only 1.3% had PJI. No comorbidity was significantly related to PJI. General anesthesia was used in almost 21% of the patients and was significantly associated with a higher risk of infection (*p* = 0.049). An increased operative time was also significantly related to PJI (*p* = 0.012). Conversely, tranexamic acid (TXA) administration was a protective factor against PJI (*p* = 0.017).

**Conclusion:**

Although PJI is not a common complication of TJA, multiple risk factors such as general anesthesia and prolonged operative time play a significant role in its development. In contrast, TXA administration is thought to reduce the risk of PJI effectively.

## Background

Total joint arthroplasty (TJA) aims to relieve pain symptoms and restore the joint function in patients with severe arthritis. Periprosthetic joint infection (PJI) is defined as postoperative infection in joint prostheses, occurring at a rate of 0.25–2.0% [1, 2]. Although TJA has been found to improve the overall quality of life of patients with end-stage arthritis, variable complications can potentially occur, jeopardizing procedural outcomes and compromising patients’ health status [3]. Even with the various advancements and improvements in joint prostheses, the incidence of PJI is increasing worldwide [4].

The increasing incidence of PJI can be thought to be related to that of comorbidities in many operative candidates. Owing to the increased life expectancy, there is an assumed future healthcare burden caused by the increasing number of osteoarthritis cases [5]. Consequently, the number of TJA that must be performed is also expected to rise. With the assumed increasing numbers, the incidence of PJI could increase proportionately in the upcoming decades [6, 7]. Based on the significance and the associated healthcare burden of PJI, the potential risk factors of this condition should be identified for early detection.

PJI is influenced by different factors that ultimately increase its risk of occurrence. These factors may include the characteristics of patients undergoing TJA, technique used during the procedure, adequacy of the postoperative period and care provided [8]. Our study aimed to review cases of TJA performed and assess the incidence of PJI and its associated factors.

## Methods

A retrospective study was conducted at King Abdulaziz Medical City (KAMC), a tertiary hospital in Riyadh. All patients of both sexes who underwent TJA from 2012 to 2022 were included. Patients who were aged under 18 years, were immunocompromised, had a history of infection and had missing data were excluded. Patients were recruited via non-probability consecutive sampling.

Data were collected through the BESTCare system at KAMC. The main categories of the data collection sheet used included the following: demographic data, operative data, comorbidities and infection. The operative data included the anesthesia type, operation name, unilaterality or bilaterality, approach, tourniquet usage, estimated blood loss amount, operative period, estimated operative time, time to postoperative infection, drainage and tranexamic acid (TXA) administration.

The presence of infection was based on the 2013 International Consensus Meeting and modified Musculoskeletal Infection Society consensus-based criteria. These criteria included the presence of two periprosthetic positive culture findings with phenotypically identical organism or sinus tract communicating with the joint or the presence of three out of the following five minor criteria: (1) elevated serum C-reactive protein level (more than 10 mg/L) and erythrocyte sedimentation rate (more than 30 mm/h); (2) elevated synovial fluid white blood cell count (more than 3000 cells/μL or++ change on a leukocyte esterase test strip); (3) elevated synovial fluid polymorphonuclear neutrophil percentage (more than 80%); (4) favorable histological finding of periprosthetic tissue; and (5) one positive culture finding.

The data were entered into Microsoft Excel (Microsoft, Redmond, WA, USA) and transferred to the Statistical Package for the Social Sciences v.25 (IBM Corp., Armonk, NY, USA) for statistical analysis. The data were checked for missing information, and new variables were recorded and computed based on the extracted data. All assumptions were satisfied for each analysis. The data analysis comprised two stages. The first stage was a descriptive analysis, wherein categorical variables were described as frequencies and percentages and numerical variables as means and standard deviations. The second stage was a bivariate analysis conducted using the Chi-squared test and Mann–Whitney test. Each analysis was performed at a confidence level of 95% and a *p* value of < 0.05.

Consent was not required for this retrospective cohort study. No identifying data were asked, ensuring privacy and confidentiality. All data were kept safe, with only the authors having access to the research data.

## Results

A total of 1198 adult patients were admitted for TJA at KAMC, Riyadh. Most of them were women (76.6%). The average age of the patients was 63 (range: 19–91) years. The majority were obese (78.4%), followed by overweight (17.9%). The patient demographics are further detailed in Table 1.

**Table 1:** Demographics

Characteristic (*n* = 1198)	*n*	(%)
*Gender*		
Men	280	23.4
Women	918	76.6
*Age*		
Less than 30	9	.8
30 to 44	28	2.3
45 to 59	362	30.2
60 to 74	639	53.3
75 years and above	160	13.4
*BMI*		
Normal weight	44	3.7
Overweight	215	17.9
Obese	939	78.4

All values are presented as numbers and percentages

Approximately 94.7% underwent total knee arthroplasty (TKA), while the rest underwent total hip arthroplasty (THA). Regarding the anesthesia utilized, the most common type was spinal anesthesia (38.3%), followed by spinal/epidural anesthesia (30%). General anesthesia was used in 21.6% of the patients and epidural anesthesia in almost 9%. Moreover, the approach used in the patients who underwent TKA was either medial parapatellar or subvastus, with most patients undergoing medial parapatellar TKA. Among the patients who underwent THA, either the posterior or lateral approach was used, with most patients undergoing posterior THA. The operative period and estimated operative time were also analyzed, showing that the majority of the procedures were performed in the morning and the minority at night, with a mean operative time of 137.9 ± 56.02 min. In addition, drainage was applied in almost 75% of the patients for a mean duration of 2.72 days. TXA was administered in 36.2% of the patients. Finally, 15 patients had PJI; of them, almost 1.3% (*n* = 5) had acute PJI, while the rest had chronic PJI. The details are shown in Table 2. The incidence of PJI was 1.3% (Table 3).

**Table 2:** Surgery details

Characteristic (*n* = 1198)	*n*	(%)
*Anesthesia type*		
Epidural	112	9.3
General	259	21.6
Spine	459	38.3
Spine/epidural	368	30.7
*Operation name*		
Total knee arthroplasty (TKA)	1135	94.7
Total hip arthroplasty (THA)	63	5.3
*Unilateral/bilateral*		
Unilateral	607	50.7
Unilateral—revision	8	.7
Bilateral simultaneous	166	13.9
Bilateral staged	417	34.8
*Approach*		
Medial parapatellar	898	75.0
Subvastus	237	19.8
Posterior	58	4.8
Lateral	5	.4
*Tourniquet*		
Applied	1071	89.4
Not applied	127	10.6
*Blood loss / mL (mean* = *235.24, SD* = *152.48)*		
*Operative period*		
Morning	748	62.4
Afternoon	408	34.1
Evening	38	3.2
Night	4	.3
*Estimated operative time / min (mean* = *137.90, SD* = *56.02)*		
*Time of PJI post-op/day*		
No	1183	98.7
Acute PJI (< 90 days)	5	.4
Chronic PJI (> 90 days)	10	.8
*Drain/no drain*		
Drain	898	75.0
No drain	300	25.0
*Duration of drain/day (mean* = *2.72, SD* = *1.77)*		
*Tranexamic acid use*		
Yes	434	36.2
No	764	63.8

All values presented as numbers and percentages for categories, mean and standard deviation for numeric

**Table 3:** PJI rate

Characteristic (*n* = 1198)	*n*	(%)
*PJI*		
Yes	15	1.3
No	1183	98.7

All values are presented as numbers and percentages

The comorbidities of the patients were also studied. Diabetes was found in almost 60% of the patients, hypertension in almost 56% and hyperlipidemia in almost 47%. Other comorbidities are mentioned in Table 4.

**Table 4:** Comorbidities

Characteristic (*n* = 1198)	Yes *n* (%)	No *n* (%)
Asthma	76 (6.3)	1122 (93.7)
Cancer	62 (5.2)	1136 (94.8)
Depression	56 (4.7)	1142 (95.3)
Diabetes	731 (61.0)	467 (39.0)
Heart failure	27 (2.3)	1171 (97.7)
Hyperlipidemia	562 (46.9)	636 (53.1)
Hypertension	675 (56.3)	523 (43.7)
Non-alcoholic fatty liver disease (NAFLD)	10 (0.8)	1188 (99.2)
Renal disease	32 (2.7)	1166 (97.3)
Stroke	22 (1.8)	1176 (98.2)

All values are presented as numbers and percentages

The Chi-square test and Mann–Whitney test were used to evaluate the relationship between PJI and the study variables. Table 5 displays the relationship between PJI and the demographic factors. No significant association was found between PJI and all demographic factors (*p* > 0.05). Table 6 shows the relationship between PJI and the operative details. There was a significant association observed between PJI and general anesthesia and staged bilateral surgery (both *p* < 0.05). Conversely, TXA administration was found to be associated with a reduced risk of PJI (*p* < 0.05). Furthermore, there was a significant relationship noted between PJI and the estimated operative time (*p* < 0.05). Table 7 displays the association between PJI and comorbidities. No significant association was found between them.

**Table 5:** Test the relation between PJI with patients’ demographic factors

Demographic factors		PJI		*P* value
		Yes *n* (%)	No *n* (%)	
Gender	Men	5 (33.3)	275 (23.2)	0.379
	Women	10 (66.7)	908 (76.8)	
Age	Less than 30	0 (0.0)	9 (0.8)	0.203
	30 to 44	0 (0.0)	28 (2.4)	
	45 to 59	2 (13.3)	360 (30.4)	
	60 to 74	8 (53.3)	631 (53.3)	
	75 years and above	5 (33.3)	155 (13.1)	
BMI	Normal weight	2 (13.3)	42 (3.6)	0.279
	Overweight	2 (13.3)	213 (18.0)	
	Obese	11 (73.3)	928 (78.4)	

No statistically associated at 0.05 level of significant

**Table 6:** Test the relation between PJI with surgery details

Surgery details		PJI			*P* value
		Yes *n* (%)	No *n* (%)	Mean ± SD	
Anesthesia type	Epidural	2 (13.3)	110 (9.3)	–	0.049*
	General	7 (46.7)	252 (21.3)		
	Spine	5 (33.3)	454 (38.4)		
	Spine/epidural	1 (6.7)	367 (31.0)		
Operation name	Total knee arthroplasty (TKA)	14 (93.3)	1121 (94.8)	–	0.813
	Total hip arthroplasty (THA)	1 (6.7)	62 (5.2)		
Unilateral/bilateral	Unilateral	3 (20.0)	604 (51.1)	–	0.036*
	Unilateral—revision	1 (6.7)	7 (0.6)		
	Bilateral simultaneous	4 (26.7)	162 (13.7)		
	Bilateral staged	7 (46.7)	410 (34.7)		
Approach	Medial parapatellar	13 (86.7)	885 (74.8)	–	0.520
	Subvastus	1 (6.7)	236 (19.9)		
	Posterior	1 (6.7)	57 (4.8)		
	Lateral	0 (0.0)	5 (0.4)		
Tourniquet	Applied	14 (93.3)	1057 (89.3)	–	0.596
	Not applied	1 (6.7)	126 (10.7)		
Blood loss / mL	–	433.3 ± 487.9	232.7 ± 142.2	235.24 ± 152.48	0.072
Operative period	Morning	10 (66.7)	738 (62.4)	–	0.776
	Afternoon	5 (33.3)	403 (34.1)		
	Evening	0 (0.0)	38 (3.2)		
	Night	0 (0.0)	4 (0.3)		
Estimated operative time / Min	–	207.5 ± 137.5	137.02 ± 53.8	137.9 ± 56.02	0.012*
Drain/no drain	Drain	11 (73.3)	887 (75.0)	–	0.885
	No drain	4 (26.7)	296 (25.0)		
Duration of drain/day	–	2.33 ± 1.79	2.73 ± 1.73	2.72 ± 1.77	0.453
Tranexamic acid use	Yes	1 (6.7)	433 (36.6)	–	0.017*
	No	14 (93.3)	750 (63.4)		

^*^ Significant relation at 0.05 level of significant

**Table 7:** Test the relation between PJI with comorbidities

Surgery details		PJI		*P* value
		Yes *n* (%)	No *n* (%)	
Asthma	Yes	1 (6.7)	75 (6.3)	0.959
	No	14 (93.3)	1108 (93.7)	
Cancer	Yes	0 (0.0)	62 (5.2)	0.363
	No	15 (100)	1121 (94.8)	
Depression	Yes	1 (6.7)	56 (4.6)	0.713
	No	14 (93.3)	1128 (95.4)	
Diabetes	Yes	12 (80.0)	719 (60.8)	0.129
	No	3 (20.0)	464 (39.2)	
Heart failure	Yes	1 (6.7)	26 (2.2)	0.345
	No	14 (93.3)	1157 (97.8)	
Hyperlipidemia	Yes	9 (60.0)	553 (46.7)	0.307
	No	6 (40.0)	630 (53.3)	
Hypertension	Yes	12 (80.0)	663 (56.0)	0.063
	No	3 (20.0)	520 (44.0)	
Non-alcoholic fatty liver disease (NAFLD)	Yes	0 (0.0)	10 (0.8)	0.615
	No	15 (100)	1173 (99.2)	
Renal disease	Yes	1 (6.7)	31 (2.6)	0.414
	No	14 (93.3)	1152 (97.4)	
Stroke	Yes	1 (6.7)	21 (1.8)	0.275
	No	14 (93.3)	1162 (98.2)	

^*^Statistically associated at 0.05 level of significant

## Discussion

Obesity has been found to be associated with a proportionately increased risk of other simultaneous comorbidities such as cardiovascular diseases, diabetes, decreased quality of nutritional intake, hypertension and possible mortality [9]. The risk of developing PJI is also likely to increase proportionately with an increasing body mass index [10]. In our study, we found that the body mass index was not a significant predictor of PJI (*p* = 0.279). In comparison, Jämsen et al. [11] reported that the incidence of PJI tended to increase with an increase in the body mass index; the incidence of PJI was 0.37% in patients who had a normal body mass index in comparison with 4.66% in patients who were considered morbidly obese. Conversely, Bozic et al. [12] noted that the risk of developing PJI postoperatively in association with the presence of obesity was considered significantly lower (19.0%) 1 year after TJA (*p* < 0.025).

Regarding sex, previous studies have noted that male candidates are more prone to developing PJI than female candidates [13, 14]. Other studies have reported opposite findings, claiming that women are more vulnerable to developing the infection than men [15, 16]. In our study, the women had a higher risk of developing PJI than the men (*p* = 0.379).

The age of the patients was not a significant predictor of PJI in our study. In a previous study, patients of older age were found to be more prone to developing postoperative infections owing to changes in their immunity [17]. In contrast, a multicenter study that analyzed around 623,253 joint operations reported that a younger age was more associated with developing PJI [18]. Similar to our study results, Wu et al. [19] found that patients aged between 65 and 75 years were at a higher risk of developing PJI by 3.36 folds than those aged between 45 and 65 years.

Comorbidities are risk factors that greatly influence the development of PJI [20]. In our study, no existing comorbidities were significantly associated with PJI. Diabetes was found in almost 61% of the patients, hypertension in 56% and hyperlipidemia in 47%. Different studies have shown that diabetes increases the risk of infection following TJA [11]. In an Indian study, it was found that there was a three-to-sixfold increased risk of PJI in patients with diabetes [21]. Moreover, a recent meta-analysis found that diabetes was associated with a 1.74-fold increased risk of PJI after TJA [22].

Some studies have shown that spinal anesthesia is associated with a lower risk of post-TJA infection and has some protective effects, including reduced amount of blood loss, reduced need for blood transfusion and lower incidence of hyperglycemia [23–25]. In our study, the most common anesthesia type was spinal anesthesia (38.3%), followed by spinal/epidural anesthesia (30%). General anesthesia was used in almost 21.6% and epidural anesthesia in almost 9%. Only general anesthesia was significantly associated with PJI (*p* = 0.049). Scholten et al. [26] showed similar results: Spinal anesthesia was more frequently utilized in patients. In their multivariable logistic regression analysis, general anesthesia yielded a higher risk for early PJI (odds ratio = 2.0; 95% confidence interval = 1.0–3.7).

The operative time is calculated from incision to closure of the skin. Peersman et al. [27] reported a significant association between SSI and subsequent PJI with a prolonged operative time. The recent meta-analysis conducted by Scigliano et al. [28] showed that following TJA, the risk of SSI and PJI was significant in procedures lasting ≥ 120 min compared with that in procedures lasting < 120 min. However, the risk of PJI was not significant when comparing both groups owing to limited data. Moreover, Wang et al. [29] showed that in TJA, an increased operative time was independently linked to PJI and SSI. They found that for every 20-min increase in the operative time, there was a 25% increase in the incidence of PJI. In our study, an increased operative time (mean = 137.9 ± 56.02 min) was significantly related to PJI (*p* = 0.012). The operative period was also analyzed and was divided into four groups as shown in Tables 2 and 5. No significant association was found with PJI (*p* = 0.776).

TXA may help reduce bleeding and hence infection by eliminating hematoma formation. Therefore, its administration is thought to have a protective effect against the risk of PJI [30]. The study based on an institutional database conducted by Yazdi et al. showed that TXA administration was associated with a reduced risk of PJI after primary TJA. Similarly, in our study, TXA administration was a protective factor against PJI (*p* = 0.017).

Different operative approaches are used in TJA based on multiple factors, including the surgeon’s preference and expertise. The optimum operative approach is still up for debate. For TKA, an international study reported that the outcomes at 1 week and 1 year were better with the subvastus approach than with the medial parapatellar approach, although the medial parapatellar approach was more convenient to use [31].

Regarding THA, a recent study was conducted by Aggarwal et al. [32] to assess the incidence of infection between different hip approaches. The authors divided the approaches into either direct anterior or non-anterior approaches and found 2.2-fold higher odds of infection in patients for whom the anterior approach was utilized. In our study, the most common approach used for TKA was the medial parapatellar approach (79%). The posterior approach was more commonly utilized than the lateral approach in almost 92% of the patients. Nevertheless, the approach was not significantly associated with the development of PJI (*p* = 0.52).

PJI is defined as infection in joint prostheses or surrounding tissues. The incidence of PJI has been found to range from 0.25 to 2.0%, indicating rarity of the infection [1]. Our study found that the risk of infection was around 1.3%. Fifteen patients had PJI, who were then divided into two groups: those with acute PJI and those with chronic PJI at a cutoff duration of 90 days. Approximately 33.3% had acute PJI, whereas the rest had chronic PJI. The recent study conducted by Kim et al. [33] showed that the annual incidence of PJI in Korea was 2.3–2.8%, comparable to 2–2.7% in the USA. The study conducted by Jin et al. [34] showed that the prevalence of PJI in four hospitals in Australia was 1.7% within 2 years following TJA. Similarly but to a lesser extent, 0.8% of the infected cases analyzed in our study were considered chronic PJI.

Postoperative drainage is a well-established standard treatment for orthopedic procedures. It is regarded as a valuable practice but is debatable. Parker et al. [35] demonstrated that operative wound drainage after orthopedic operations was associated with decreased wound discharges, which may minimize the risks of prosthetic infections later. A meta-analysis conducted in China also showed that the use of wound drainage resulted in an increased risk of PJI [36]. In our study, 75% of the patients had postoperative drainage for a mean duration of 2.72 days. Nevertheless, there was no significant association between the use of drainage and the incidence of PJI (*p* = 0.885).

Tourniquet use during TKA has long been considered to reduce blood loss amount, improve visualization and facilitate operation. However, the medical community has debated upon its role in the risk of PJI. In our study, tourniquet was applied in 89.4% of the patients. There was no significant association between the usage of tourniquet and the risk of PJI (*p* = 0.596). In their meta-analysis, Zhang et al. [37] concluded that there was no significant difference in the incidence of PJI between patients who underwent operation with and without tourniquet use. However, the recent meta-analysis by Magan et al. [38] noted that using tourniquet in TKA was associated with an increased risk of infection, prolonged length of stay and increased need for blood transfusion.

TKA can be performed in multiple ways, including simultaneous, staged bilateral or unilateral TKA. In the study conducted by Poultsides et al. [39], the incidence of widespread infection in simultaneous bilateral TKA (0.57%) was significantly lower than that in bilateral staged TKA (1.39%) and unilateral TKA (1.1%). Similarly, in our study, there was a significant association between staged bilateral TKA and the incidence of PJI (*p* = 0.036), representing 46.7% of the PJI cases. Hart et al. [40] showed that simultaneous bilateral TKA was generally safe and did not lead to more hospitalizations than did unilateral TKA. Another meta-analysis conducted by Xu et al. [41] reported that there was no increased risk of PJI following simultaneous bilateral TJA when compared with staged bilateral TJA.

Regarding the limitations of this study, the data were gathered from only one center, potentially restricting the generalizability of the findings. This subject requires more exploration in the area, with a larger sample size involved. In addition, owing to the retrospective study design, there was a limitation in the ability to control for the variables, which might have influenced the results. Moreover, owing to the limited number of studies that investigated the predictors of PJI, we faced difficulties in comparing the results.

## Conclusion

PJI is not a common complication of TJA, but risk factors such as general anesthesia and prolonged operative time can lead to its development. Conversely, TXA administration can reduce the risk of PJI. The modifiable risk factors of PJI should be acknowledged and optimized to provide optimum healthcare to patients after TJA.

## Data Availability

All data generated or analyzed during this study are included in this published article.

## Abbreviations

TJA: Total joint arthroplasty
PJI: Periprosthetic joint infection
KAMC: King Abdulaziz Medical City
TXA: Tranexamic acid
TKA: Total knee arthroplasty
THA: Total hip arthroplasty
